# Hispanics and Hospice Care: A Systematic Review

**DOI:** 10.1093/geroni/igaa057.1056

**Published:** 2020-12-16

**Authors:** Valeria Cardenas, Susan Enguidanos, Gillian Fennell

**Affiliations:** University of Southern California, Los Angeles, California, United States

## Abstract

Hospice care has demonstrated improved pain and symptom relief for patients at end-of-life, however, Hispanics have significantly lower rates of hospice use compared to Whites. Moreover, few studies have examined factors associated with these lower enrollment rates and barriers to hospice care experienced by Hispanics. This systematic literature review aims to provide a comprehensive overview of studies examining Hispanic hospice use. We conducted a comprehensive search using three electronic databases (Ovid Medline, EMBASE, and CINAHL) from January 1946 to March 2019 using MESH terms for Hispanics, hospice, and end-of-life care. Our review was guided by the Preferred Reporting Items for Systematic Reviews and Meta-Analysis Protocols (PRISMA-P). Studies of Hispanic adults living in the United States that examined hospice use, outcomes of care, or knowledge and attitudes towards hospice were included. Commentaries, case studies, editorials, literature reviews were excluded. Of the 4,230 abstracts reviewed, 43 peer-reviewed articles met the inclusion criteria. Among these studies, barriers to hospice among Hispanics included lack of hospice knowledge and awareness, language barriers, and cultural barriers. Among most studies, Hispanics were less likely to receive hospice care than Whites, although some studies found that among those that enrolled in hospice, Hispanics had longer lengths of stay than whites. Overall few studies examined Hispanics use of hospice, and among those we found most were of moderate and low quality. More research is needed to understand the full range of Hispanics experiences of hospice care. Such research could guide efforts to develop culturally tailored care for this community.

